# Oxygen Glucose Deprivation Induced Prosurvival Autophagy Is Insufficient to Rescue Endothelial Function

**DOI:** 10.3389/fphys.2020.533683

**Published:** 2020-09-16

**Authors:** Venkateswaran Natarajan, Tania Mah, Chen Peishi, Shu Yi Tan, Ritu Chawla, Thiruma Valavan Arumugam, Adaikalavan Ramasamy, Karthik Mallilankaraman

**Affiliations:** ^1^Mitochondrial Physiology and Metabolism Lab, Department of Physiology, Yong Loo Lin School of Medicine, National University of Singapore, Singapore, Singapore; ^2^Department of Physiology, Anatomy and Microbiology School of Life Sciences, La Trobe University, Melbourne, VIC, Australia; ^3^Genome Institute of Singapore, A*STAR, Singapore, Singapore; ^4^Center for Healthy Longevity, NUHS, Singapore, Singapore

**Keywords:** MCUR1, endothelial dysfunction, oxygen-glucose deprivation, autophagy, apoptotic cell death

## Abstract

Endothelial dysfunction, referring to a disturbance in the vascular homeostasis, has been implicated in many disease conditions including ischemic/reperfusion injury and atherosclerosis. Endothelial mitochondria have been increasingly recognized as a regulator of calcium homeostasis which has implications in the execution of diverse cellular events and energy production. The mitochondrial calcium uniporter complex through which calcium enters the mitochondria is composed of several proteins, including the pore-forming subunit MCU and its regulators MCUR1, MICU1, and MICU2. Mitochondrial calcium overload leads to opening of MPTP (mitochondrial permeability transition pore) and results in apoptotic cell death. Whereas, blockage of calcium entry into the mitochondria results in reduced ATP production thereby activates AMPK-mediated pro-survival autophagy. Here, we investigated the expression of mitochondrial calcium uniporter complex components (MCU, MCUR1, MICU1, and MICU2), induction of autophagy and apoptotic cell death in endothelial cells in response to oxygen-glucose deprivation. Human pulmonary microvascular endothelial cells (HPMVECs) were subjected to oxygen-glucose deprivation (OGD) at 3-h timepoints up to 12 h. Interestingly, except MCUR1 which was significantly downregulated, all other components of the uniporter (MCU, MICU1, and MICU2) remained unchanged. MCUR1 downregulation has been shown to activate AMPK mediated pro-survival autophagy. Similarly, MCUR1 downregulation in response to OGD resulted in AMPK phosphorylation and LC3 processing indicating the activation of pro-survival autophagy. Despite the activation of autophagy, OGD induced Caspase-mediated apoptotic cell death. Blockade of autophagy did not reduce OGD-induced apoptotic cell death whereas serum starvation conferred enough cellular and functional protection. In conclusion, the autophagic flux induced by MCUR1 downregulation in response to OGD is insufficient in protecting endothelial cells from undergoing apoptotic cell death and requires enhancement of autophagic flux by additional means such as serum starvation.

## Introduction

Endothelial cells are essential regulators of vascular function. Endothelial dysfunction is widely implicated in the development and progression of many vascular diseases (Deedwania, 2003; Gutierrez et al., 2013). Endothelial mitochondria plays an important role as a key regulator of endothelial function (Groschner et al., 2012). Defects in mitochondrial function could potentially contribute to development and progression of endothelial dysfunction (Tang et al., 2014). Endothelial mitochondria, beyond its role in providing support in energy production, also aid in shaping cytosolic Ca^2+^ signals and redox regulation (Dedkova and Blatter, 2005; Wilson et al., 2019). Ca^2+^ is an important second messenger that determines both cellular bioenergetics and the initiation of cell death mechanisms (Groschner et al., 2012; Williams et al., 2013; Natarajan et al., 2020). Mitochondrial matrix calcium regulates important cofactors for enzymes involved in the Krebs cycle – namely pyruvate dehydrogenase, isocitrate dehydrogenase and α-ketoglutarate dehydrogenase (Mallilankaraman et al., 2012a; Tarasov et al., 2012; Vatrinet et al., 2017). These enzymes are essential players of Krebs cycle which provides reducing equivalents to the electron transport chain (ETC), thereby contributing to the majority of mitochondrial ATP production (Quijano et al., 2016). Although the role of oxidative phosphorylation as an energy source in endothelial cells remains questionable (Quintero et al., 2006), it is still a major source of ROS (Murphy, 2009) that contributes to the pathophysiology of many cardiovascular diseases (Madamanchi and Runge, 2007). Furthermore, mitochondrial Ca^2+^ signaling has been shown to regulate NO production in endothelial cells (Williams et al., 2013; Park and Park, 2015).

Ca^2+^ enters the mitochondria through a highly calcium selective ion channel, the mitochondrial calcium uniporter (Kirichok et al., 2004). Mitochondrial calcium uniporter is a multiprotein complex comprising of, the pore forming subunit MCU (Baughman et al., 2011; De Stefani et al., 2011; Chaudhuri et al., 2013), regulatory subunits MCUR1 (Mallilankaraman et al., 2012a; Vais et al., 2015), MICU1 (Perocchi et al., 2010; Mallilankaraman et al., 2012b; Logan et al., 2014), MICU2 (Payne et al., 2017), MICU3 (Patron et al., 2019), MCUb (Raffaello et al., 2013) and EMRE (Sancak et al., 2013; Vais et al., 2016; Payne et al., 2020), of which MCU, MCUR1, MICU1, and MICU2 are well characterized. Despite the discovery of the components of mitochondrial calcium uniporter in the last decade, the role of these components in endothelial mitochondrial dysfunction during ischemic vascular injury remains poorly understood.

Mitochondrial calcium overload has been implicated in endothelial cells subjected to ischemic stress leading to deregulated NO signaling and ROS production (Choy et al., 2001). Mitochondrial Ca^2+^ overload and ROS overproduction are known to trigger the opening of mitochondrial permeability transition pore (mPTP), a large non-selective pore that spans across the IMM and OMM, leading to cell death (Bernardi, 1999; Duchen, 2000). On the other hand, loss of calcium transfer to mitochondria or defects in mitochondrial calcium uptake leads to ATP depletion resulting in activation of AMPK-mediated macro autophagy (Cardenas et al., 2010; Mallilankaraman et al., 2012a).

Here, we employed human pulmonary endothelial cells subjected to oxygen-glucose deprivation (OGD) to study the status of mitochondrial calcium uniporter components. Our results suggest that MCUR1 alone is downregulated under OGD conditions, whereas the other uniporter components tested namely MCU, MICU1 and MICU2 remains unchanged. Downregulation of MCUR1 activated pro-survival autophagy, which was still insufficient to rescue endothelial function. Blockade of autophagic flux did not confer protection against OGD-induced cell death, indicating a need for additional autophagy inducers to enhance autophagic flux. In the current study, we show the modulation of mitochondrial calcium uniporter and its downstream autophagic induction is insufficient and requires additional induction of autophagy to rescue endothelial function under OGD conditions.

## Materials and Methods

### Cell Line

Wild type Human Pulmonary Micro Vascular Endothelial Cells (HPMVECs) were grown in low glucose Dulbecco’s modified Eagle’s medium (DMEM) supplemented with 10% fetal bovine serum (FBS), 100 U/ml penicillin, and 100 μg/ml streptomycin at 37°C and 5% CO_2_.

### Oxygen-Glucose-Deprivation (OGD) Treatment

Wild type HPMVECs were seeded at a density of 3.0 × 10^6^ cells/plate in 10 cm plates 1 day before the experiments. Media in wild type HPMVECs were replaced with glucose-free Locke’s buffer (154 mM NaCl, 5.6 mM KCl, 2.3 mM CaCl_2_, 1 mM NaHCO_3_ 5 mM HEPES at pH 7.2, supplemented with 5 mg/L gentamicin) and incubated in oxygen-deprivation chamber (Billups-Rothenberg, San Diego, CA, United States). For oxygen deprivation the chambers were flushed with a gaseous mix of 95% Nitrogen (N_2_) and 5% CO_2_ for 10 min. The oxygen-deprivation chamber was sealed and kept in an incubator at 37°C. Controls were incubated with low glucose DMEM supplemented with 10% FBS, 100 U/ml penicillin, and 100 μg/ml streptomycin at 37°C and 5% CO_2_. The length of incubation in oxygen-glucose deprived conditions were 3, 6, 9, and 12 h, and referred to as OGD3, OGD6, OGD9, and OGD12, respectively. For chloroquine (CQ) or Metformin (Met) treatment studies, CQ or Met was added into the glucose-free Locke’s buffer prior to incubation of the cells for 3, 6, 9, or 12 h of OGD. Controls were treated with CQ or Met 3 h prior before collection. For serum starvation studies, the cells were grown with 10% serum for 24 h. The medium was removed, washed and incubated with low glucose DMEM supplemented with 0.2% serum 24 h prior to OGD. Controls were incubated with low glucose DMEM supplemented with 0.2% serum throughout the experiment until cell lysate collection.

### Cell Lysate Preparation

Following OGD treatments, cell culture dishes were kept on ice, the cells scraped and collected into tubes. The cell pellets were then washed with cold PBS and centrifuged at 1500 × *g* for 5 min at 4°C. Cells were lysed by resuspending the pellet with RIPA lysis buffer (Thermo Scientific, #89900) containing protease inhibitor and phosphatase inhibitor cocktail (Thermo Scientific, #1860932). After sonication, cell lysates were centrifuged at 13,000 × *g* for 15 min at 4°C and the collected supernatant from each sample was stored at −80°C. Total protein concentration present in the collected supernatant was quantified using the Thermo Scientific Pierce^TM^ bicinchoninic acid (BCA) Protein Assay Kit (Thermo Scientific, #23225). Each sample containing 30 μg of protein was denatured by boiling at 95°C for 10 min in 2 X Laemmli buffer (Bio-Rad Laboratories, #1610737) and β-mercaptoethanol (Sigma Aldrich) at 1:1 ratio before being subjected to Western blot analysis.

### Western Blot

Protein samples were separated on a sodium dodecyl sulfate (SDS) polyacrylamide gel. 10% SDS-polyacrylamide gels (resolving gel: 8 ml of H_2_O, 4 ml of 40% acrylamide, 4 ml of Tris buffer pH 8.5, 150 μl of APS and 13 μl of TEMED; stacking gel: 5.75 ml of H_2_O, 0.75 ml of 40% acrylamide, 1 ml of Tris buffer pH 6.5, 77 μl of APS and 7.7 μl of TEMED) were used to separate MCU, MCUR1, MICU1, MICU2, AMPK, and phospho-AMPK (pAMPK). 15% SDS-polyacrylamide gels (resolving gel: 6 ml of H_2_O, 6 ml of 40% acrylamide, 4 ml of Tris buffer pH 8.5, 150 μl of APS and 13 μl of TEMED; stacking gel: 5.75 ml of H_2_O, 0.75 ml of 40% acrylamide, 1 ml of Tris buffer pH 6.5, 77 μl of APS and 7.7 μl of TEMED) were used to separate full-length Caspase 3, cleaved Caspase 3, and LC3B. Gel electrophoresis was run at 75 V for the first 30 min, then increased to 100 V for 90 min in 1 X Tris/Glycine/SDS running buffer (25 mM Tris, 192 mM glycine, 0.1% SDS, pH 8.3; Bio-Rad Laboratories, #1610772). The separated proteins on the gel were then transferred onto nitrocellulose membranes (Bio-Rad Laboratories, #1620112) using a wet transfer apparatus. The electroblotting was run at 350 mA for 100 min in chilled 1 X Tris/Glycine transfer buffer (25 mM Tris, 192 mM glycine, 20% (v/v) methanol, pH 8.3; Bio-Rad Laboratories, #161-0771). After transfer, membranes were subjected to blocking in 5% non-fat dry milk diluted in 1 X Tris–buffered saline containing 0.1% Tween-20 (1xTBST) for 1 h at room temperature and incubated with primary antibody overnight at 4°C with gentle shaking. Membranes were washed with 1xTBST three times, 10 min per wash, to remove excess primary antibody before incubation with horseradish peroxidase (HRP)-conjugated anti-rabbit or anti-mouse secondary antibody for 1 h at room temperature. Anti-rabbit secondary antibodies were used in the detection of MCU, MCUR1, MICU1, MICU2, AMPK, pAMPK, Caspase 3, cleaved Caspase 3, and LC3B, while anti-mouse secondary antibodies were used in the detection of β-actin. Membranes were again washed with 1xTBST three times, 10 min per wash, to remove excess secondary antibody. To visualize bands representing protein-of-interest, the membranes were incubated with chemiluminescent ECL reagent (BioRad, #1705061) for 3 min and specific bands were then detected on X-ray films (Research Instruments). To ensure equal protein loading across gels, membranes were stripped using Restore Western Blot Stripping Buffer (ThermoFisher Scientific, #21059) and probed with a loading control antibody. Antibodies for MCU (Sigma-Aldrich, HPA016480; 1:250); MCUR1 (Proteintech, 24948-1-AP; 1:500); MICU1 (Sigma-Aldrich, HPA037479; 1:500); MICU2 (Abcam, ab101465; 1:1000); Caspase 3 (Cell Signalling, 9662S; 1:1000); AMPK (Merck, 07-350; 1:1000); pAMPK (Cell Signaling, 40H9; 1:1000); LC3B (Cell Signalling, 2775S; 1:1000); anti-actin (Sigma-Aldrich, A2228; 1:5000); anti-mouse IgG-HRP (BBI Life Sciences, D110085-0100; 1:10,000); and anti-rabbit IgG-HRP (Amersham, NA934V; 1:2000) were used in the study. Relative band intensities were measured using ImageJ Imaging Software and expressed as a value normalized by the intensity of β-actin signal.

### Migration Assay

Wild type HPMVECs were seeded at a density of 0.5 × 10^6^ cells/well in six-well plates and incubated overnight at 37°C. A uniform 1.8 mm scratch running the entire length of the well was created using a sterile 200 μl tip. For the “Normal” condition, the wells were washed thrice with PBS to remove cell debris after making the scratch and then bathed in 2 ml low glucose DMEM supplemented with 10% FBS, 100 U/ml penicillin, and 100 μg/ml streptomycin. For endothelial cells subjected to 8 h of OGD, the wells were washed thrice with PBS to remove cell debris after making the scratch and then bathed in 2 ml glucose-free Locke’s Buffer. For endothelial cells subjected to 8 h of OGD with serum starvation, the cells were pre-starved overnight prior to OGD treatment. Similar to the 8 h of OGD treatment, the wells were washed thrice with PBS to remove cell debris after making the scratch and then bathed in 2 ml of glucose-free Locke’s Buffer. the cells were incubated at 37°C and 5% CO_2_. The time-lapse of the cell migration within each well was captured using EVOS live cell imaging system equipped with on-stage incubation system by capturing images every 15 min for 8 h. Similarly, for OGD conditions the imaging was performed but gas mixture was set to 95% N_2_ and 5% O_2_.

### Statistical Data Analysis

Data from multiple experiments were quantified and expressed as mean ± SEM. In order to analyze the differences between two groups, the two-tailed unpaired Student’s *t*-test was used. All experiments were repeated n number of times as indicated in figure legends. Data were computed with GraphPad Prism software version 8.0.1, where *p*-values measuring the level of significance in differences observed between two groups were obtained. Any significant difference between the two groups were then indicated by either an asterisk ^∗^ or ns, where ns represents non-significant, ^∗^represents *p* < 0.05; ^∗∗^represents *p* < 0.01; ^∗∗∗^represents *p* < 0.001 and ^****^represents *p* < 0.0001.

## Results

### Modulation of Mitochondrial Calcium Uniporter Complex in Human Pulmonary Microvascular Endothelial Cells (HPMVECs) in Response to Oxygen-Glucose Deprivation (OGD)

**FIGURE 1:**
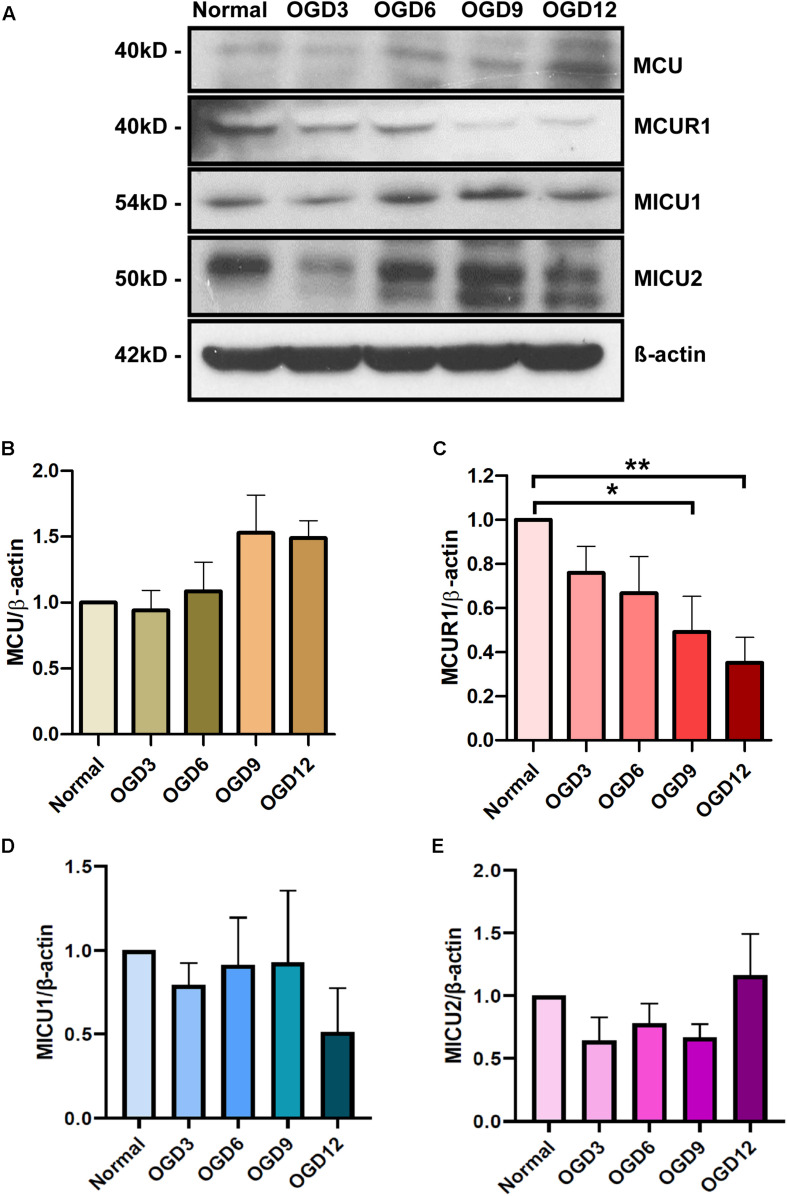
**(A)** Representative western blots and **(B–E)** quantification showing the expression levels of **(B)** MCU, **(C)** MCUR1, **(D)** MICU1, and **(E)** MICU2 in human endothelial cells under in vitro OGD conditions at different time points as indicated. β-action was used as a loading control. mean ± SEM, *n* = 5, ns = non-significant; *P < 0.05; **P < 0.01.

To determine the expression pattern of the mitochondrial calcium uniporter complex components in HPMVECs in normal and OGD conditions, protein levels of MCU, MCUR1, MICU1, and MICU2 were assessed by western blot analysis (Figure 1A). Quantitative analyses showed no significant changes in MCU (Figure 1B), MICU1 (Figure 1D), and MICU2 (Figure 1E) expression across the OGD timepoints, but significant reduction in MCUR1 (Figure 1C) levels at 12 h of OGD. These findings suggest that OGD results in downregulation of MCUR1, the positive regulator of the mitochondrial calcium uniporter complex.

### OGD Induces Endothelial Cell Death Despite the Induction of Autophagic Flux

**FIGURE 2:**
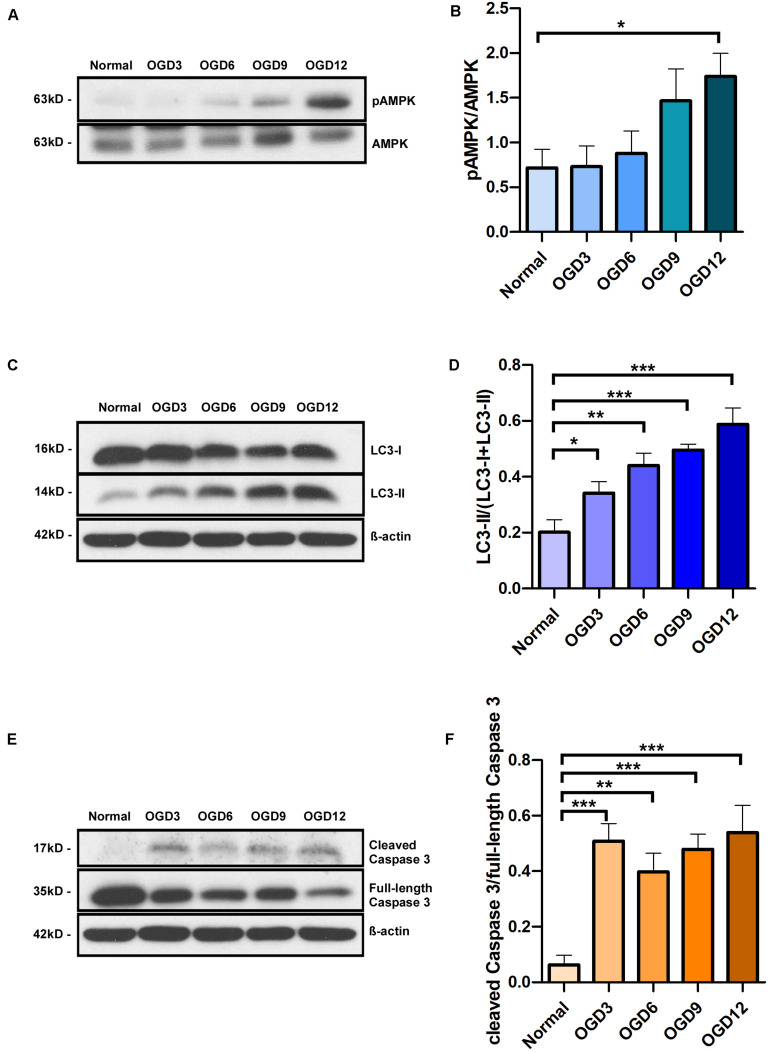
Representative western blots and quantification of **(A,B)** Phospho-AMPK and total AMPK, **(C,D)** LC3-I and LC3-II, in human endothelial cells, **(E,F)** cleaved Caspase 3 and full-length Caspase 3 under in vitro OGD conditions at different time points as indicated. β-action was used as a loading control. mean ± SEM, *n* = 4; *P < 0.05; **P < 0.01; ***P < 0.001.

MCUR1 downregulation is known to activate pro-survival autophagy (Mallilankaraman et al., 2012a). Since we observed a decrease in MCUR1 levels following OGD (Figures 1A,C), we sought to verify the status of autophagic flux. As expected, significant increase in autophagic markers phospho-AMPK (pAMPK)/AMPK ratio and LC3 processing was observed, which correlates with the significant decrease in MCUR1 observed over the same duration of OGD (Figures 2A–D). OGD has also been known to cause cell death in many cell types including endothelial cells (Xu et al., 2000; Plesnila et al., 2001). Therefore, we wanted to determine the expression level of apoptotic cell death markers during OGD. We observed significant increase in cleaved caspase 3 during OGD (Figures 2E,F), suggesting the activation of apoptotic cell death. Altogether, this suggests that despite the induction of autophagy which plays a protective role, HPMVECs still undergo apoptotic cell death induced by OGD.

### Blockade of Autophagic Flux Has no Effect on OGD-Induced Cell Death

**FIGURE 3:**
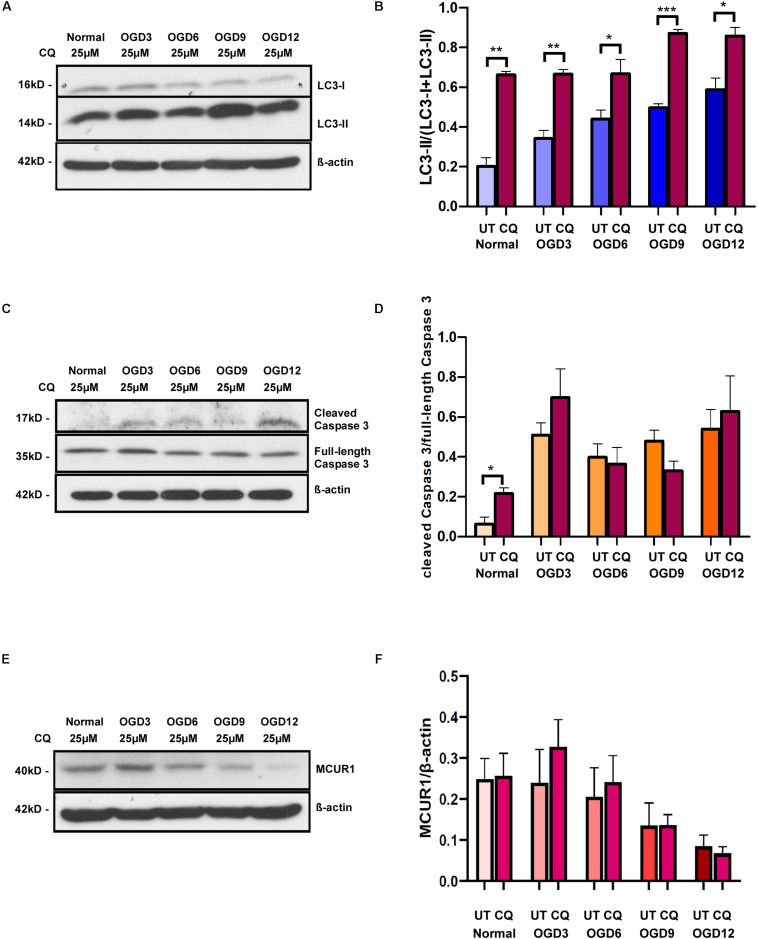
Representative western blots and quantification showing the expression levels of **(A,B)** LC3-I and LC3-II **(C,D)** cleaved Caspase 3 and full-length Caspase 3 **(E,F)** MCUR1 in human endothelial cells under in vitro OGD conditions with (+) or without (−) chloroquine (CQ) at different time points as indicated. β-action was used as a loading control. mean ± SEM, *n* = 4; *P < 0.05; **P < 0.01; ***P < 0.001.

Autophagy is known to have a close connection with caspase-mediated cell death, despite its well accepted pro-survival role. To verify whether the observed OGD-induced cell death is triggered by autophagy, we blocked the autophagic flux using a widely used inhibitor Chloroquine (CQ) and assessed the cell death under different OGD conditions. Chloroquine inhibits autophagosome-lysosome fusion and there by enhances accumulation of LC3-II. Cells treated with CQ showed an increased LC3-II accumulation compared to untreated ones in both normoxic and OGD conditions (Figures 3A,B). Interestingly, CQ treatment did not alter OGD-induced caspase-mediated cell death (Figures 3C,D). Chloroquine has been reported to have additional effects on mitochondrial function and whether it affects MCUR1 expression remains unstudied. Like untreated cells, MCUR1 was significantly reduced in CQ-treated HPMVECs over 12 h of OGD (Figures 3E,F) suggesting CQ has no effect on MCUR1 expression. Taken together, these data suggest that autophagy induction is not responsible for caspase-mediated cell death in OGD conditions.

### Serum Starvation Attenuates OGD-Induced Cell Death

Autophagic flux in OGD conditions neither have any protective effects nor causes cell death. Therefore, we speculated insufficient autophagic flux as a cause for lack of protection wherein enhancement of autophagic flux could rescue the cells from OGD-induced cell death. For induction of higher autophagic flux in endothelial cells under hypoxic conditions, we attempted using a previously reported activator of autophagy Metformin. However, good autophagic induction was not observed after treatment of cells with Metformin (Supplementary Figure 1), possibly due to the similar route these drugs take to activate autophagy as OGD (Meng et al., 2015; Kim et al., 2016). While Metformin failed to protect the cells from cell death at 3 and 6-h post OGD, it significantly reduced cell death at OGD 9 and 12 h (Supplementary Figure 1). This could possibly due to delayed induction of autophagy. Therefore we sought to attempt serum starvation, a widely used potent physiological inducer of autophagy (Mizushima and Klionsky, 2007; Mizushima et al., 2010). A recent study has shown short term serum starvation (12–48 h) induced autophagy via Akt-mTOR-p70S6K inhibition offered protection against endothelial blood brain barrier impairment (Yang et al., 2019). Therefore, we attempted to test the protective effect of serum starvation induced autophagy against OGD induced cell death.

**FIGURE 4:**
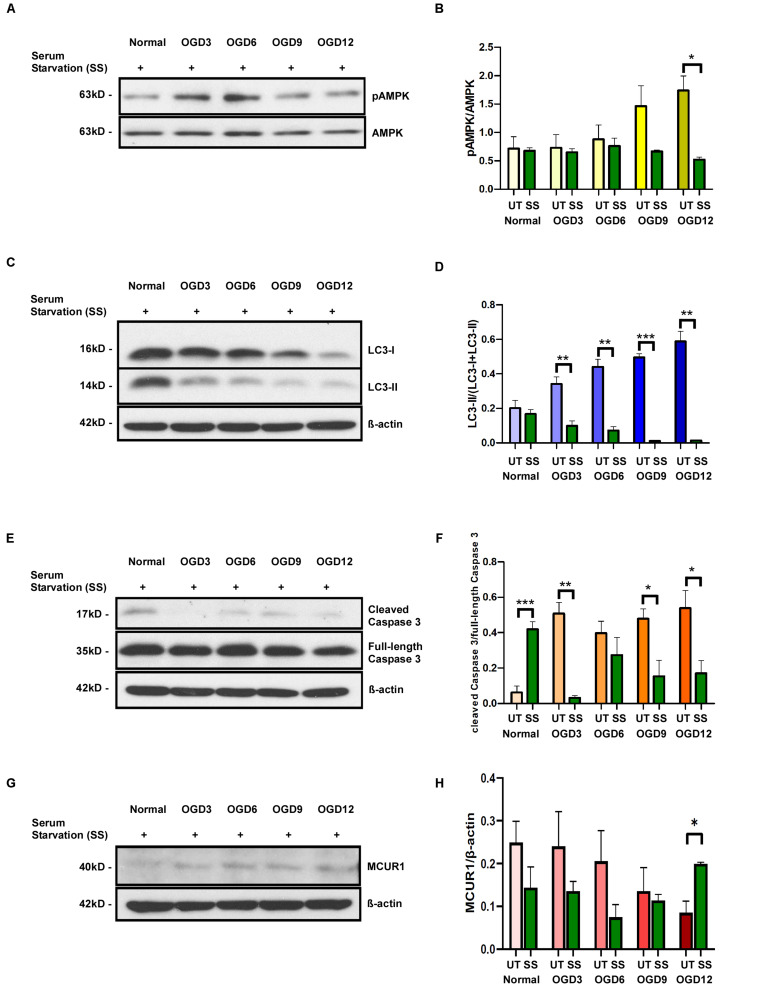
Representative western blots and quantification of **(A,B)** Phospho-AMPK and total AMPK, **(C,D)** LC3-I and LC3-II **(E,F)** cleaved Caspase 3 and full-length Caspase 3, **(G,H)** MCUR1 in serum-starved in human endothelial cells under in vitro OGD conditions at different time points as indicated. β-action was used as a loading control. mean ± SEM, *n* = 4; *P < 0.05; **P < 0.01; ***P < 0.001.

Cells that were serum-starved for 24 h before OGD treatment showed increased levels of pAMPK/AMPK ratio and LC3 processing under normoxic conditions indicating an increase in autophagic flux. But the pAMPK/AMPK ratio in serum-starved cells did not increase during OGD (Figures 4A,B). Interestingly, LC3-II/I ratio decreased over different time points of OGD (Figures 4C,D) indicating the degradation of autophagosomes as seen in the final stages of autophagic process. To verify whether the serum starvation-induced enhancement of autophagic flux has any protective role during OGD, we assessed the Caspase 3 cleavage. The ratio of cleaved caspase 3 to full-length caspase 3 was significantly reduced in serum-starved group compared to during OGD (Figures 4E,F). Interestingly, Serum starvation was able to rescue the MCUR1 expression at OGD 12 (Figures 4G,H). Overall, these data suggest that serum starvation mediated enhanced induction of autophagy serves as a mechanism that confers protection of HPMVECs against cell death under OGD stress. To understand whether serum starvation-induced increase in autophagic flux is due to further decrease in MCUR1 during OGD, we verified the MCUR1 expression in serum-starved cells undergoing OGD treatment. Surprisingly, except OGD12 time point in which MCUR1 expression was increased, all other time points did not have significant change in MCUR1 expression levels indicating the increase in autophagic flux during serum starvation was independent of MCUR1 pathway (Figures 4G,H).

### Serum Starvation Rescues Endothelial Migration Despite the OGD Treatment

**FIGURE 5:**
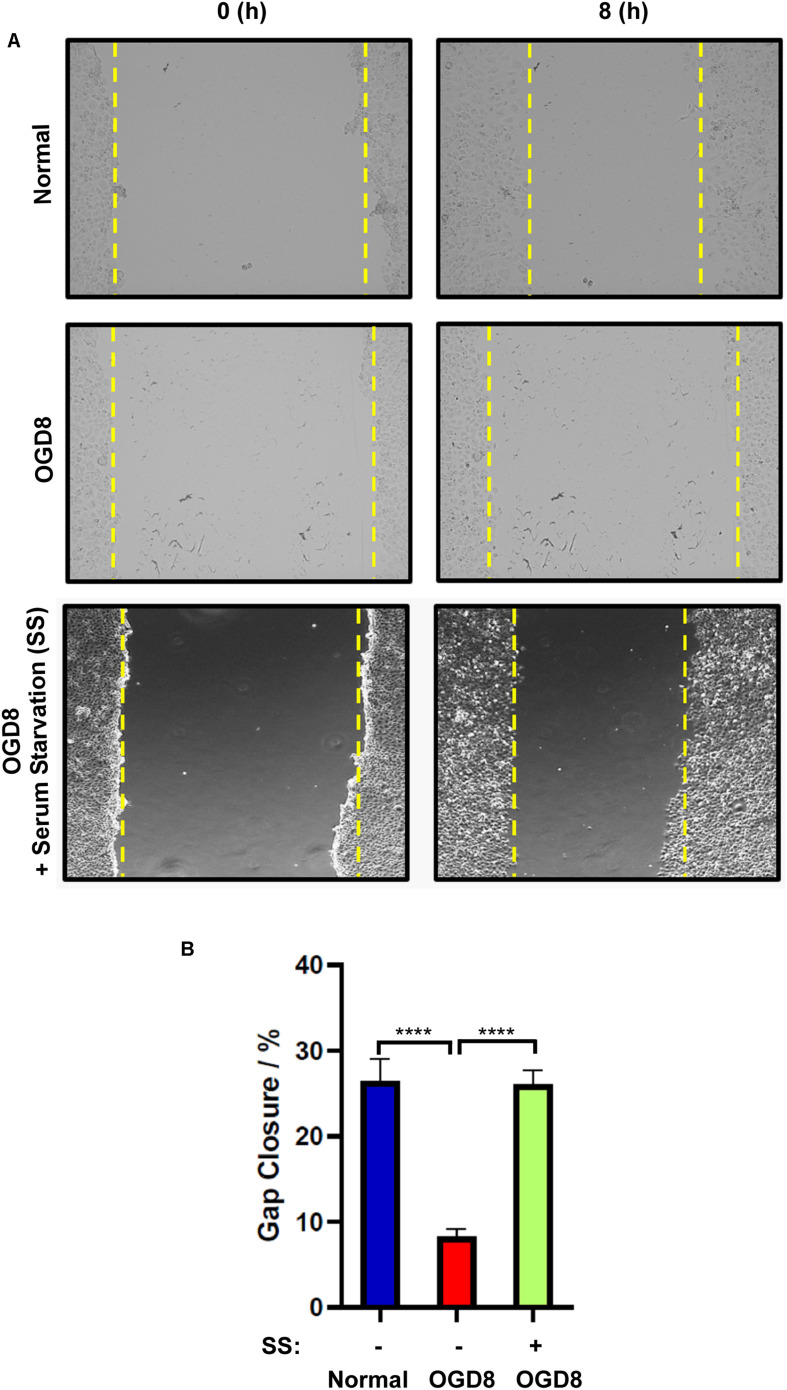
**(A)** Representative images and **(B)** quantification of endothelial cell migration and normal, or OGD conditions with (+) or without (–) serum-starvation 8 h post-scratch using EVOS live cell imaging system. mean ± SEM, *n* = 4; ****P < 0.0001.

To assess the functional status of endothelial cells under OGD conditions, we employed a widely used *in vitro* wound-healing scratch assay which implies the migratory potential. Endothelial cells migrated to cover the scratch area under normoxic conditions. However, this migration was significantly reduced under OGD condition. Since serum starvation had effectively reduced OGD-induced cell death, we sought to understand whether this could rescue the endothelial function. Interestingly, serum-starved endothelial cells under OGD conditions migrated similar to cells under normoxic conditions (Figures 5A,B). Overall, these data indicate that the serum starvation-induced autophagic flux rescues endothelial function despite OGD treatment.

## Discussion

Ischemic injury causes endothelial dysfunction leading to functional decline of the vasculature. Role of endothelial mitochondria in response to ischemic insults have long been known but the underlying mechanisms are poorly understood. Here, we show MCUR1, a positive regulator of mitochondrial calcium uniporter is downregulated in response to *in vitro* ischemic conditions leading to activation of AMPK-mediated pro survival autophagy. Despite this autophagic flux, endothelial cells continue to activate apoptotic cell death suggesting a distinct pathway that surpasses pro survival autophagy. Nonetheless, activation of autophagic flux by serum starvation confers protection against OGD-induced endothelial cell death. Therefore, targeting mitochondrial calcium uniporter components to activate autophagy is insufficient in protecting from ischemic injury and requires a stronger inducer of autophagy. Our study clarifies the level of autophagic flux determining the protective function in ischemic injury.

Mitochondrial calcium overload has been implicated in ischemic injury of many cell types. Nonetheless, the status of the major ion channel through which calcium enters the mitochondria remains mysterious. This study shows the modulation of the positive regulator of mitochondrial calcium uniporter, MCUR1 (Figure 1), suggesting a defect in mitochondrial calcium uptake during OGD conditions. Further, the activity of calcium-dependent enzymes in the mitochondrial matrix that contributes the reducing equivalents to ETC decreases, leading to an energy crisis. Previous studies have shown that blockade of Ca^2+^ transfer from ER to mitochondria or defects in mitochondrial calcium uptake leads to activation of prosurvival autophagy (Cardenas et al., 2010). Interestingly, loss of MCUR1 has been reported to trigger the AMPK-mediated prosurvival autophagy (Mallilankaraman et al., 2012a). AMPK is well-recognized as an energy sensor that is typically activated allosterically by AMP (Langendorf et al., 2016). Reversal of AMP to ATP ratio happens during MCUR1 loss that leads to phosphorylation of AMPK (Mallilankaraman et al., 2012a). This instigated us to propose MCUR1 downregulation as a protective mechanism that sets in during OGD conditions in activating AMPK-mediated prosurvival autophagy. To further support the activation of autophagic flux data, LC3 processing was also observed in endothelial cells undergoing OGD. The increased processing of LC3-I to LC3-II, promotes the formation of autophagosomes that will eventually be degraded in the autophagic process (Tamargo-Gomez and Marino, 2018). Thus, the increase in the ratio of pAMPK to total AMPK together with the increase in LC3 processing suggests the induction of prosurvival autophagy (Figure 2). Nonetheless, our data supplements the few studies reporting OGD-induced apoptotic cell death in cells such as neurons and cerebral endothelial cells (Xu et al., 2000; Plesnila et al., 2001).

Surprisingly, caspase-mediated apoptotic cell death was increased despite the activation of prosurvival autophagy (Figure 2). Previous studies have shown AMPK-mediated autophagy as a pro-survival mechanism (Borger et al., 2008; Cardenas et al., 2010; Mallilankaraman et al., 2012a), implying that cell death should be reduced upon activation of AMPK-mediated autophagy. Whereas we observed both apoptosis and autophagic induction occurring simultaneously in endothelial cells subjected to OGD. This raised a concern whether AMPK-mediated autophagy induced in these cells under OGD stress, is serving as a protective mechanism or one that is promoting cell death. It must be noted that the paradoxical role of AMPK in autophagy and apoptotic cell death has been reported (Paz et al., 2016). While the accumulation of LC3-II demonstrates the induction of autophagy, it could also indicate the inhibition of autophagic flux, whereby autophagic flux measures the degradation activity of autophagic cargo. Therefore, we determined the autophagic flux induced in endothelial cells subjected to OGD by using a widely used inhibitor of autophagy, Chloroquine (CQ). CQ impairs the fusion of autophagosomes with lysosomes (Mauthe et al., 2018), thereby reduces the degradation of autophagic cargo, and inhibits autophagic flux (Mauthe et al., 2018). Our study showed a significant increase in LC3-II levels in the CQ-treated group compared to the corresponding untreated group, suggesting that the accumulation of LC3-II over time of OGD in untreated cells is not due to inhibition of autophagic flux, but rather a flux that is occurring at low and inefficient levels. Furthermore, since LC3-II is a marker of autophagosomes, significantly higher level of LC3-II in CQ-treated cells under normoxic conditions also indicates the accumulation of autophagosomes and thus the successful blockade of autophagic flux by CQ (Figure 3).

While the autophagic flux is inhibited by CQ treatment, OGD-induced cell death in CQ-treated group remained similar to untreated group (Figure 3). This suggests that the induction of AMPK-mediated autophagy in endothelial cells under OGD stress is playing a protective role as inhibition of pro-death autophagy should have reduced cell death. Moreover, the MCUR1 levels in both the untreated and CQ-treated groups remain unchanged suggesting that the downregulation of MCUR1 is upstream of autophagic flux. These data suggest that the endogenous induction of autophagic flux is insufficient in conferring protection against OGD-induced apoptotic cell death. The extent of autophagic flux seems to be the factor deciding whether cells survive or undergo apoptotic cell death. Therefore, we attempted to increase the autophagic flux in endothelial cells by other means and assess the degree of protection against OGD-induced cell death. Attempts with chemical activators such as Metformin induced autophagy like untreated group. However, Metformin reduced cell death only at 9 and 12 h of OGD compared untreated group (Supplementary Figure 1). This delay in protection could be due to the similarity in pathways that OGD and the chemical activators use to activate autophagy (Meng et al., 2015; Kim et al., 2016). Therefore, we decided to attempt serum starvation, which is a widely used and potent physiological inducer of autophagy (Mizushima and Klionsky, 2007; Mizushima et al., 2010). Further, Serum starvation was recently shown to induce autophagy in endothelial cells via Akt-mTOR-p70S6K pathway.

Serum-starved cells showed significantly reduced pAMPK levels indicating the cell’s attempt to prevent excessive and constitutive AMPK activation, which could induce a “metabolic failure-like” state (Ramamurthy and Ronnett, 2006). While this finding suggests an increase in autophagic flux in serum-starved endothelial cells under OGD stress, the decrease in LC3-II levels further supports the enhancement of autophagic flux. LC3-II, a marker of autophagosomes, is expected to decrease as autophagosomes get degraded in the final stages of the autophagic process (Tamargo-Gomez and Marino, 2018). Interestingly, serum starvation-induced increase in autophagic flux correlated with significant decrease in caspase-mediated cell death, suggesting that the increase in autophagic flux could offer protection against OGD-induced cell death. An increase in autophagic flux by serum starvation in OGD conditions increases the amino acid availability through efficient degradation of autophagic cargo, thereby increasing protein synthesis (Herzig and Shaw, 2018). These amino-acids as well as the fatty acids liberated from bulk autophagic degradation can be recycled in a cell autonomous fashion and utilized by TCA cycle to maintain ATP production (Kuma et al., 2004; Lum et al., 2005; Mizushima, 2007; Mizushima and Klionsky, 2007). To further support the serum starvation-induced enhancement of autophagic flux, MCUR1 levels were rescued despite the OGD conditions in serum-starved endothelial cells presumably from the amino acids generated by autophagic degradation.

Endothelial cells migrate during angiogenesis, but they do migrate under pathophysiological conditions to restore vascular integrity (Michaelis, 2014). The ability of endothelial cells to migrate requires an enhancement in glycolysis, a process which is greatly reduced in cells subjected to OGD (Diebold et al., 2019). Besides requiring energy to drive the migration of endothelial cells, the role of mitochondrial Ca^2+^ homeostasis has been recently implicated in actin cytoskeleton dynamics and cell migration in mammals (Prudent et al., 2016). Specifically, endothelial cell migration is regulated by intracellular Ca^2+^ signaling, whereby Ca^2+^ acts on cytoskeleton architecture, migration direction, and focal adhesion dynamics (Prudent et al., 2016). We also observed defective migration in cells undergoing OGD. This is most likely attributed to the compromised energy production as the glycolytic process is less efficient in producing ATP in the absence of exogenous glucose supply. In addition, the reduced mitochondrial Ca^2+^ uptake resulting from MCUR1 downregulation is unable to sufficiently fuel oxidative phosphorylation to generate ATP. Interestingly, serum starvation rescued the defects in migration caused by OGD suggesting that serum starvation-induced autophagic flux rescued the migratory ability of the endothelial cells under OGD. Serum starvation induced autophagic degradation liberates amino acids and fatty acids that fuels the TCA cycle for energy production. The energy required for the endothelial migration is derived from the autophagic degradation and thereby rescues OGD induced impairment of endothelial migration. Further, restoration of MCUR1 expression by serum starvation could have rescued the mitochondrial Ca^2+^ uptake which inturn is essential for ATP production (Glitsch et al., 2002; Prudent et al., 2016).

Overall, our study identified perturbation in expression of MCUR1, the positive regulator of mitochondrial calcium uniporter in OGD conditions, resulting in the activation of pro-survival autophagy. While the autophagic flux that normally occurs during OGD conditions are insufficient to confer protection against apoptotic cell death, serum starvation induces autophagy and multiple pathways to offer protection and restore endothelial function.

## Conclusion

**FIGURE 6:**
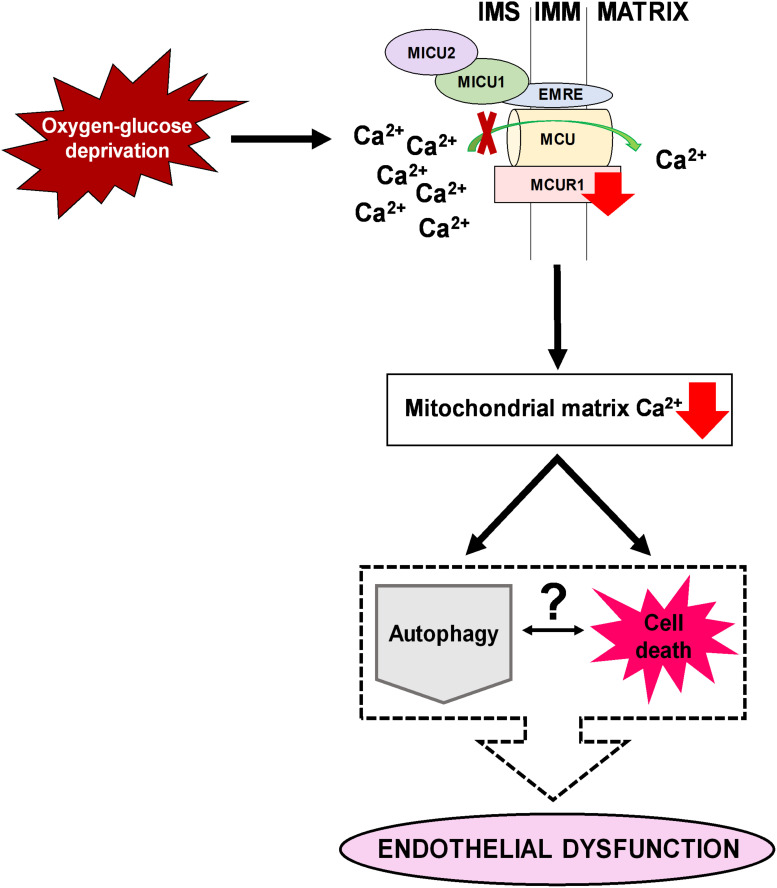
A schematic diagram illustrating an overview of how downregulation of MCUR1 reduces mitochondrial calcium uptake in human endothelial cells subjected to OGD. While pro-survival AMPK-mediated autophagy was induced under OGD stress, cell death was observed to be occurring simultaneously thus questioning the relationship between autophagy and cell death in contributing to the development of endothelial dysfunction.

In conclusion, our study has demonstrated the downregulation of mitochondrial calcium uniporter component MCUR1, leading to activation of both autophagic flux and OGD induced endothelial cell death. Careful analysis showed that the induced autophagic flux due to MCUR1 loss was insufficient to offer protection and required additional activators of autophagy to protect from cell death and rescue endothelial function (Figure 6). Serum starvation, a potent physiological activator of autophagy enhanced the autophagic flux, reversed the loss of MCUR1, and rescued endothelial function despite the presence of OGD stress. Our study suggests that targeting the mitochondrial calcium uniporter components in ischemic vascular diseases does not confer sufficient protection against endothelial dysfunction.

## Data Availability Statement

All datasets generated for this study are included in the article/Supplementary Material.

## Author Contributions

TM, VN, and KM participated in the design of the study. TM, VN, CP, RC, and KM conducted the experiments. AR, TV, and KM contributed with reagents or analytical tools. CP and ST prepared reagents for the study. TM, VN, CP, and KM performed the data analysis. AR performed the statistical analysis. TM, VN, and KM wrote the manuscript. All authors discussed and reviewed the manuscript.

## Conflict of Interest

The authors declare that the research was conducted in the absence of any commercial or financial relationships that could be construed as a potential conflict of interest.
