# Supplementary figures and images for: Oxygen Glucose Deprivation Induced Prosurvival Autophagy Is Insufficient to Rescue Endothelial Function
Source: Front Physiol. 2020 Sep 16;11:533683. doi: 10.3389/fphys.2020.533683 (PMC7526687; doi:10.3389/fphys.2020.533683)

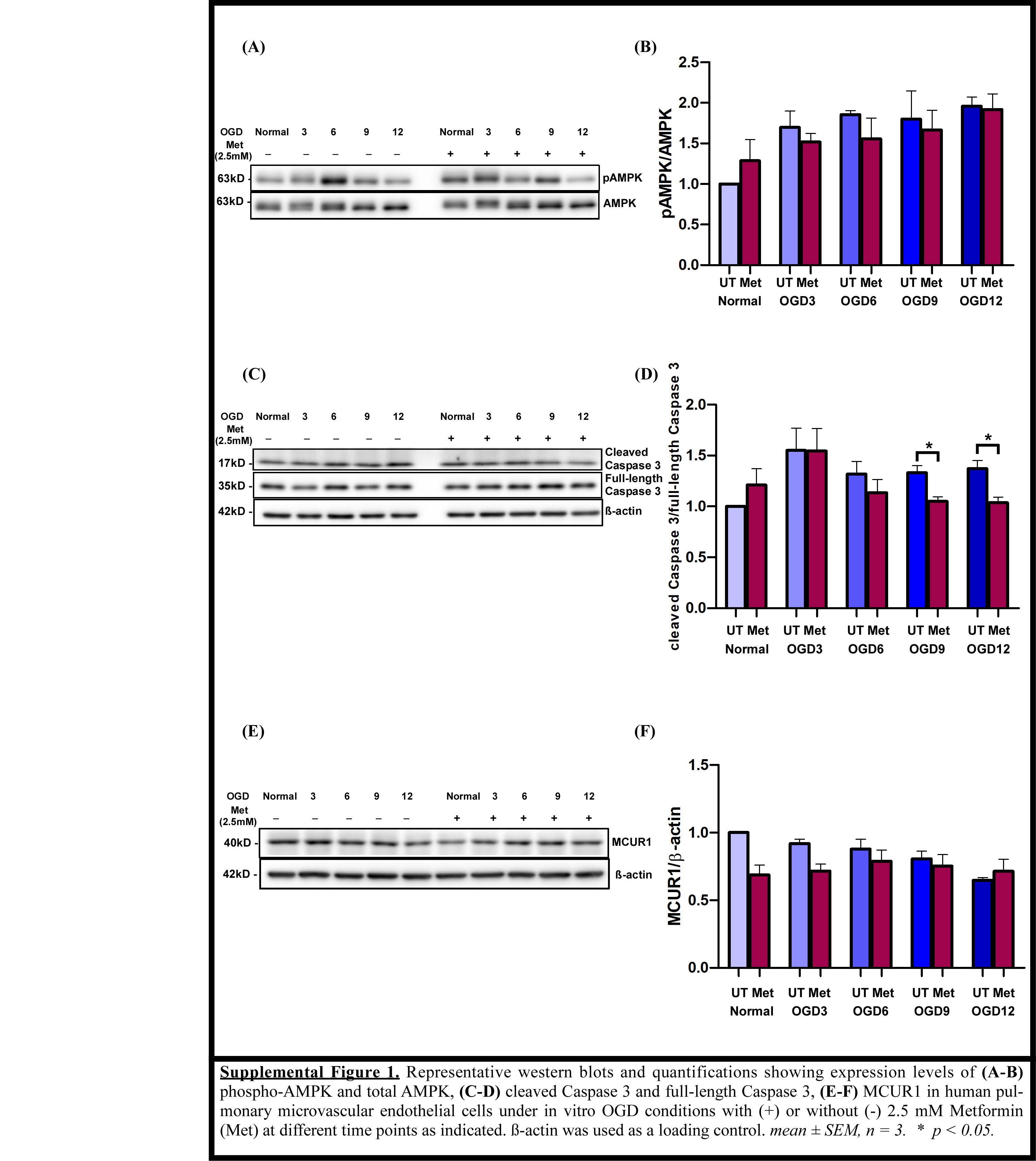

Supplement: Supplementary file 1 [file Image_1.tif]
